# Supplementary material for: Identification of Freezing-Responsive microRNAs and Their Targets in Chinese Jujube by Small RNA and Degradome Sequencing
Source: Int J Mol Sci. 2026 Sep 4;27(17):7912. doi: 10.3390/ijms27177912 (PMC13566890; doi:10.3390/ijms27177912)
Supplement: Supplementary file 1 [file ijms-27-07912-s001.zip › Supplementary Material S2/novel_miR_144.pdf]

Provisional ID	:	novel_miR_144
Score total	:	31.4
Score for star read(s)	:	3.9
Score for read counts	:	29.7
Score for mfe	:	0
Score for randfold	:	-2.2
Score for cons. seed	:	
Total read count	:	70
Mature read count	:	58
Loop read count	:	9
Star read count	:	3

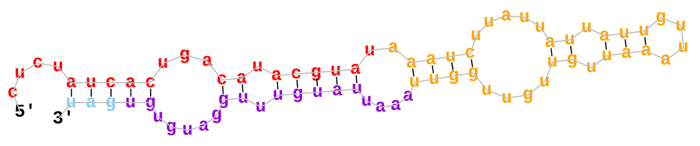

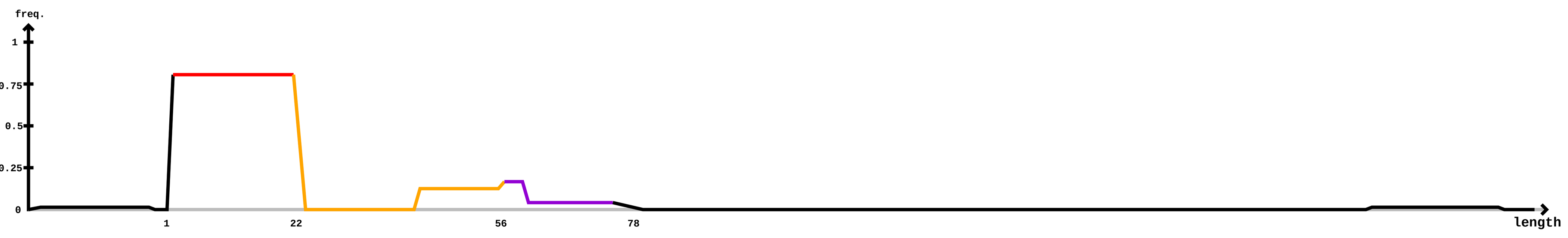

Mature Star

```
5'- auauuugaguucuagguuuuuucucuaucacugacauacguauaaaucuuauuauuauuguuaaauuguuguugguuaaauuauguuuggaugugugauuauauuuagaaccaaaaaaaaugaaaaaaaaaacaaaacccaaaaaucaaacaaacaaacuuuuggaugugugg caccuuaauuucauacuauuuguaaguggguuuguaccaauaaguauccagauacuguaagucacuuuggaauauuu -3'   obs
    auauuugaguucuagguuuuuucucuaucacugacauacguauaaaucuuauuauuauuguuaaauuguuguugguuaaauuauguuuggaugugugauuauauuuagaaccaaaaaaaaugaaaaaaaaaacaaaacccaaaaaucaaacaaacaaacuuuuggaugugugg caccuuaauuucauacuauuuguaaguggguuuguaccaauaaguauccagauacuguaagucacuuuggaauauuu        exp
    ...((((.((((((((((.(..((.(((((.(.((((((......((((.....((.(((....))).))....))))....)))))).))))).).))..).))))))))))))).................................((((((..((((.((((((((((((.........)))))).))))).))))..))))))......((((((((((.(((...)))...))))).))))))   reads   mm   sample
    auCuuugaguucuagguuu........................................................................................................................................................................................   1   1   LT-3
    ......................cucuaucacugacauacguau................................................................................................................................................................   30   0   LCK-1
    ......................cucuaucacugacauacguau................................................................................................................................................................   28   0   LCK-2
    ..........................................................aauuguuguugguuaaau...............................................................................................................................   9   0   LCK-3
    .........................................................................aaaGuauguuuggaugugu..............................................................................................................   3   1   LT-2
    .......................................................................................................................................................................cagauacuguaagucacuuugg.......   1   0   LT-3
```
